# Gastroschisis at school age: what do parents report?

**DOI:** 10.1007/s00431-019-03417-5

**Published:** 2019-07-19

**Authors:** Annelieke Hijkoop, André B. Rietman, René M. H. Wijnen, Dick Tibboel, Titia E. Cohen-Overbeek, Joost van Rosmalen, Hanneke IJsselstijn

**Affiliations:** 1 Department of Pediatric Surgery and Intensive Care, Erasmus MC–Sophia Children’s Hospital, Room SP-3506, P.O. Box 2060, 3000 CB Rotterdam, The Netherlands; 2 Department of Child and Adolescent Psychiatry/Psychology, Erasmus MC–Sophia Children’s Hospital, Rotterdam, The Netherlands; 3 Department of Obstetrics and Gynecology, Division of Obstetrics and Prenatal Medicine, Erasmus MC–Sophia Children’s Hospital, Rotterdam, The Netherlands; 4 Department of Biostatistics, Erasmus MC, Rotterdam, The Netherlands

**Keywords:** Gastroschisis, Abdominal wall defect, Outcome, Behavior, Cognition, Quality of life

## Abstract

Children with gastroschisis are at high risk of morbidity in early life, which could affect long-term outcomes. We determined parent-reported outcomes in school-aged children born in 2000–2012, using paper questionnaires. Parent-perceived child vulnerability and motor function were compared with the Dutch reference data; parent-rated data on cognition, health status, quality of life, and behavior were compared with those of controls matched for age, gender, and maternal education level. Of 77 eligible participants, 31 (40%) returned the questionnaires. Parent-reported motor function was normal in 23 (74%) children. Total scores on health status, quality of life, and behavior did not differ significantly from those of matched controls. Children with gastroschisis had lower scores on cognition (median (interquartile range); 109 (87–127)) than their matched controls (124 (113–140); *p* = 0.04). Neonatal intestinal failure and increased parent-perceived vulnerability were associated with lower scores on cognition (β − 25.66 (95% confidence interval − 49.41, − 1.91); − 2.76 (− 5.27, − 0.25), respectively).

*Conclusion*: Parent-reported outcomes of school-aged children with gastroschisis were mainly reassuring. Clinicians and parents should be aware of the higher risk of cognitive problems, especially in those with neonatal intestinal failure or increased parent-perceived vulnerability. We recommend multidisciplinary follow-up at school age of children with gastroschisis and neonatal intestinal failure.

**Table:** 

**What is Known:** • Many infants with gastroschisis experience morbidity in early life. • Data on developmental outcomes and daily functioning in children with gastroschisis beyond the age of 5 years are scarce and conflicting.	
**What is New:** • Parents of school-aged children treated for gastroschisis report normal motor function, health status, quality of life, and behavior. • Children with gastroschisis, especially those with intestinal failure, may be at risk for cognitive problems at school age. Parents who reported their child as being more vulnerable also reported more cognitive problems at school age.

**Electronic supplementary material:**

The online version of this article (10.1007/s00431-019-03417-5) contains supplementary material, which is available to authorized users.

## Introduction

Gastroschisis is a life-threatening congenital abdominal wall defect requiring surgical treatment shortly after birth. Nowadays, over 90% of cases are diagnosed prenatally [1], which allows for early parental counseling. Additional anomalies are relatively rare, and survival rates are over 90% [2]. However, these infants are at high risk of morbidity, especially those with associated intestinal defects (complex gastroschisis [3]). Morbidities include intestinal failure, prolonged length of hospital stay (LOS), and complications such as adhesive small bowel obstruction, parenteral nutrition-related cholestasis, and sepsis [4–7]. In addition to having undergone surgery in early life, many of these infants are born small for gestational age (SGA) [8, 9] or preterm [2], which may affect neurodevelopmental outcomes [10–13]. Parent-reported outcome measures are becoming increasingly relevant, but data on outcomes at school age are scarce and conflicting [14–18].

To optimize follow-up and to improve parental counseling, we evaluated parent-reported motor function, cognition, health status, quality of life, and behavior in school-aged children (i.e., 4–17 years) with gastroschisis. In addition, we sought to identify predictors of cognition and behavior at school age, including parent-perceived child vulnerability, infant clinical data, sociodemographic characteristics, and neurodevelopmental outcomes that had been evaluated in these children at 2 years of age [7].

## Materials and methods

### Participants

We sent paper questionnaires with a self-addressed envelope to the caregivers of all surviving children born with gastroschisis between 2000 and 2012, and treated at our hospital. Questionnaires were sent once. In non-responders, a follow-up phone call was made after 2 to 4 weeks to check whether the questionnaires had been received. These caregivers had been offered to enter their child in the longitudinal prospective follow-up program that since 1999 is standard of care for children with anatomical congenital anomalies treated at our hospital [19]. Based on the favorable outcomes reported previously [15, 19], the follow-up duration of children born with gastroschisis was limited to 2 years. Those with intestinal failure were invited to join an intestinal rehabilitation program.

**Fig. 1:**
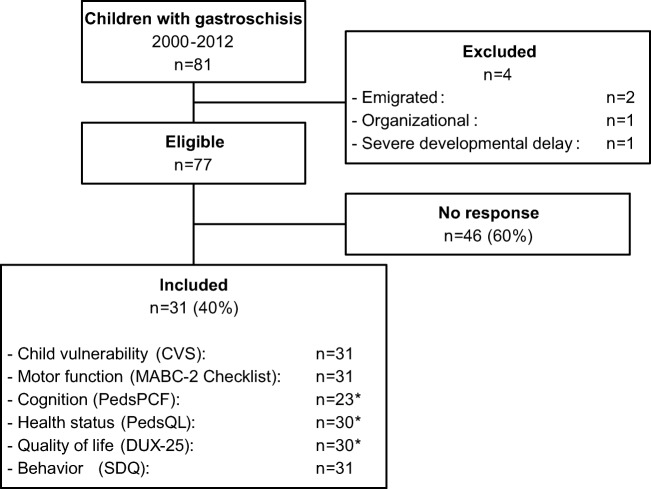
Inclusion flow chart. *Reasons for missing data: *cognition* (*n* = 8): child aged < 7 years (n = 8); *health status* (*n* = 1): questionnaire missing (*n* = 1); *quality of life* (*n* = 1): excluded because of > 3 missing values (*n* = 1).

At 2 years of age, the children’s mental and motor development had been assessed using the Bayley Developmental Scales [20] or, from December 2003, the Bayley Scales of Infant Development-Second edition [21]. Both tests provide a psychomotor and mental developmental index (mean score 100, SD 15). Neurodevelopmental outcomes at 2 years of age in those with prenatally diagnosed gastroschisis have been published previously [7]. For the purpose of the current study, we excluded four children (Fig. 1). The Medical Ethical Review Board waived approval (‘Medical Research in Human Subjects Act does not apply to this research proposal’).

### Data collection

We retrieved infant clinical data from medical records. Preterm birth was defined as delivery < 37 weeks of gestation. Infants with a birth weight < 10th centile for Dutch reference curves were classified as SGA [22]. Those with additional intestinal defects (i.e., atresia, volvulus, necrosis, or perforation) were diagnosed with complex gastroschisis. We documented multiple congenital anomalies (MCA) that required surgery or multiple follow-up visits. If the time to full enteral feeding (TFEF) exceeded 2 years, the duration was set at 730 days. Intestinal failure was defined as TFEF > 6 weeks.

Socioeconomic status (SES) scores (population mean 0, SD 1) were based on postal codes at birth [23, 24]. The child’s living situation, medical data, and educational information were retrieved from a background questionnaire (Online Resource 2). Maternal and paternal education level were classified according to the International Standard Classification of Education (ISCED) 2011, with ISCED 0–2 considered as low, ISCED 3–4 as middle, and ISCED 5–8 as high [25].

### Measures

We assessed the following outcome measures from parent-reported questionnaires (Dutch versions). A detailed description of each questionnaire is provided in Online Resource 2. For the analyses of cognition, health status, quality of life, and behavior, for each child with gastroschisis, we included two controls matched for age (maximum difference of 1 year), gender, and maternal education level (low, middle, or high [25]). Matched controls were randomly selected from three recently collected datasets for different outcome measures (Online Resource 3).

#### Child vulnerability

Child Vulnerability Scale (CVS).

#### Motor function

Movement Assessment Battery for Children-Second Edition (MABC-2) Checklist.

#### Cognition

Parents of children aged ≥ 7 years rated cognitive functioning via the Pediatric Perceived Cognitive Function (PedsPCF) questionnaire.

#### Health status and quality of life

Pediatric Quality of Life Inventory (PedsQL; health status) and DUX-25 (quality of life). As no matched controls were available for DUX-25 scores in 4–7 year-olds, these data were analyzed separately.

#### Behavior

Strengths and Difficulties Questionnaire (SDQ).

### Statistical analysis

Continuous variables are presented as median (IQR), and categorical variables as number (%). Baseline characteristics of responders and non-responders were compared using Mann-Whitney tests (continuous variables), and chi-square or Fisher’s exact tests (categorical variables). One-sample Wilcoxon signed-rank tests served to compare median scores of participants with those reported in the reference population; Mann-Whitney U tests and chi-square or Fisher’s exact tests served to compare PedsPCF, PedsQL, DUX-25, and SDQ scores between participants and their matched controls.

To find possible predictors of cognition and behavior at school age, we used univariable linear regression analyses. These included parent-perceived child vulnerability, infant clinical data, sociodemographic characteristics, and neurodevelopmental outcomes at 2 years of age. Results were considered significant at *p* < 0.05.

## Results

Of 77 eligible participants, 31 (40%) caregivers returned the questionnaires (Fig. 1). Children of responders had a significantly higher SES score, were less often born SGA, and had shorter LOS than children of non-responders (Table 1).

**Table 1:** Infant clinical data and sociodemographic data of responders (*n* = 31) and non-responders (*n* = 46)

	*n*	Responders *n* = 31 (40%)	*n*	Non-responders *n* = 46 (60%)	*p* value
Age at current study (years)	31	9 (6–13)	46	9 (6–11)	0.38
Infant clinical data					
Prenatal diagnosis	31	27 (87%)	46	44 (96%)	0.21
Intoxications during pregnancy	24		43		
- Alcohol		–		–	n/a
- Smoking		11 (46%)		17 (40%)	0.62
- Recreational drugs		3 (13%)		2 (5%)	0.34
Male sex	31	17 (55%)	46	17 (37%)	0.12
Gestational age at birth (weeks)	31	37.0 (36.0–37.4)	46	36.4 (34.5–37.5)	0.39
Preterm birth	31	14 (45%)	46	27 (59%)	0.24
Birth weight (grams)	31	2500 (2200–2910)	46	2310 (2026–2663)	0.09
Small for gestational age	31	2 (6%)	45	12 (27%)	0.03
Complex gastroschisis	31	3 (10%)	46	8 (17%)	0.51
Primary closure	31	23 (74%)	46	31 (67%)	0.52
Multiple congenital anomalies^a^	31	4 (13%)	46	1 (2%)	0.15
Number of procedures under general anesthesia	31	2 (1–3)	46	2 (1–3)	0.24
Duration of initial mechanical ventilation (days)	29	2 (1–6)	46	2 (1–9)	0.49
Sepsis	31	10 (32%)	46	28 (61%)	0.02
Length of hospital stay (days)	31	35 (22–45)	46	50 (30–88)	0.02
Time to full enteral feeding (days)	31	25 (17–40)	45	36 (21–75)	0.06
Intestinal failure	31	7 (23%)	45	19 (42%)	0.08
- Time to full enteral feeding (days)	7	61 (48–67)	19	92 (64–159)	0.14
Sociodemographic data					
Maternal age at conception (years)	26	26.6 (20.6–30.9)	44	22.2 (19.7–27.4)	0.10
Socioeconomic status score	31	0.00 (−0.60 to 0.43)	46	−0.41 (−1.86 to 0.33)	0.04
- Low status score (< − 1)	31	5 (16%)	46	21 (46%)	0.01
Maternal education level	30			n/a	
- Low (ISCED 0–2)		7 (23%)			
- Middle (ISCED 3–4)		15 (50%)			
- High (ISCED 5–8)		8 (27%)			
Paternal education level	24			n/a	
- Low (ISCED 0–2)		8 (33%)			
- Middle (ISCED 3–4)		12 (50%)			
- High (ISCED 5–8)		4 (17%)			
Two caregivers at home	31	23 (74%)		n/a	
Primary language at home: Dutch	31	31 (100%)		n/a	
Neurodevelopmental data at 2 years					
Mental developmental index^b^	25	101 (94–108)	28	101 (90–112)	0.90
- Delayed (< 85)	25	4 (16%)	28	4 (14%)	1.00
Psychomotor developmental index^c^	20	91 (87–97)	27	94 (89–102)	0.37
- Delayed (< 85)	20	4 (20%)	27	6 (22%)	1.00

Data are presented as median (IQR) or *n* (%). *ISCED* International Standard Classification of Education

^a^Responders: polydactyly (*n* = 2), cryptorchidism (*n* = 1), hypospadias (*n* = 1); non-responders: urethral valves (*n* = 1)

^b^Missing data responders: organizational (*n* = 4), non-cooperative child (*n* = 1), parental refusal (*n* = 1); missing data non-responders: organizational (*n* = 4), non-cooperative child (*n* = 1), parental refusal (*n* = 12), migration (*n* = 1)

^c^Missing data responders: organizational (*n* = 6), non-cooperative child (*n* = 4), parental refusal (*n* = 1); missing data non-responders: organizational (*n* = 5), non-cooperative child (*n* = 1), parental refusal (*n* = 12), migration (*n* = 1)

### Background

Participating children had a median age of 9 years (IQR 6–13; range 4–16). Twenty-eight (90%) were raised by at least one biological parent, and three (10%) lived in a foster family. Twenty-three (74%) children had two caregivers at home. The questionnaires were answered by either the child’s mother (*n* = 22), both parents (*n* = 6), or a foster parent (*n* = 3).

Seven (23%) of 30 children required medication; one parent did not answer this question. Medication was prescribed for gastro-intestinal problems (*n* = 5), attention deficit hyperactivity disorder (ADHD; *n* = 1), or ADHD with an anxiety disorder (*n* = 1). Eleven (35%) parents reported that their child had behavioral or emotional problems, such as ADHD, autism, anxiety, or aggression. Five (16%) children attended special education; all five were reported to have behavioral or emotional problems.

### Child vulnerability

The CVS score of children with gastroschisis (median 2 (IQR 0–5)) was significantly higher than that of the reference population (i.e., median CVS: 1 [26], *p* = 0.004). Three (9%) children were perceived as being highly vulnerable; all had simple gastroschisis without MCA.

### Motor function

MABC-2 Checklist scores were available for all 31 children (Fig. 1). Twenty-three (74%) scored within the normal range, four (13%) had borderline scores, and four (13%) were highly likely to have motor problems. One of these latter eight children had complex gastroschisis, none had MCA. Ball skills were particularly problematic.

### Cognition

PedsPCF scores were analyzed in all 23 children aged 7 years or older. Their PedsPCF score (median 109 (IQR 87–127)) was significantly lower than that of matched controls (124 (113–140), *p* = 0.04; Table 2). The proportion of children scoring ≤ − 1 SD was significantly higher in the gastroschisis group (10/23, 43%) than in matched controls (5/46, 11%, *p* = 0.002). Of the three children with complex gastroschisis, two scored ≤ − 1 SD.

**Table 2:** Cognition, health status, quality of life, and behavior of children with gastroschisis compared with control groups

	**Gastroschisis** ^a^ *n* = 23	**Matched control group** *n* = 46	***p*** **value**
**Cognition (PedsPCF)**			
Total score	109 (87–127)	124 (113–140)	0.04
	**Gastroschisis** ^a^ *n* = 30	**Matched control group** *n* = 60	***p*** **value**
**Health status (PedsQL)**			
Total score	86 (72–90)	84 (74–93)	0.82
- Physical functioning	92 (84–100)	91 (81–99)	0.42
- Emotional functioning	80 (64–86)	75 (61–89)	0.93
- Social functioning	85 (74–100)	90 (75–100)	0.50
- School functioning	78 (59–90)	80 (70–99)	0.04
**Quality of life (DUX-25)**			
Total score (4–7 years old); *n* = 12	85 (76–97)	n/a	n/a
- Physical functioning	88 (72–99)		
- Emotional functioning	88 (76–100)		
- Social functioning	80 (71–96)		
- Home functioning	90 (76–100)		
Total score (8–17 years old); *n* = 18	74 (64–95)	85 (75–93)	0.12
- Physical functioning	67 (58–94)	88 (75–96)	0.03
- Emotional functioning	73 (56–88)	82 (71–93)	0.19
- Social functioning	79 (67–90)	84 (69–93)	0.36
- Home functioning	78 (64–100)	93 (80–100)	0.04
	**Gastroschisis** ^a^ *n* = 31	**Matched control group** *n* = 62	***p*** **value**
**Behavior (SDQ)**			
Total difficulties score	10 (4–14)	6 (3–10)	0.15
- Emotional problems	2 (0–3)	1 (0–3)	0.39
- Conduct problems	2 (0–3)	1 (0–2)	0.31
- Hyperactivity-inattention	4 (1–6)	3 (1–6)	0.42
- Peer problems	1 (0–2)	1 (0–1)	0.07
- Prosocial behavior	9 (7–10)	9 (8–10)	0.71

Data presented as median (IQR). *p* values were derived from Mann-Whitney U tests

^a^For one child, maternal education level was unknown. This child was matched to a control with middle maternal education level

*PedsPCF*, Pediatric Perceived Cognitive Function questionnaire; *PedsQL*, Pediatric Quality of Life Inventory; *SDQ*, Strengths and Difficulties Questionnaire

### Health status

PedsQL scores were available for 30 children. Their total score (median 86 (IQR 72–90)) was similar to that of matched controls (84 (74–93), *p* = 0.82), as well as subscale scores for physical, emotional, and social functioning (Table 2). The subscale score for school functioning was significantly lower in children with gastroschisis (median 78 (59–90) versus 80 (70–99), *p* = 0.04; Table 2).

### Quality of life

DUX-25 total scores were available for 30 children, of whom 18 were 8–17 years old. In this latter group, the difference in median DUX-25 total score between children with gastroschisis (74 (IQR 64–95)) and matched controls (85 (75–93)) did not reach statistical significance (*p* = 0.12; Table 2). Children with gastroschisis had significantly lower subscale scores for physical functioning (67 (58–94)) and home functioning (78 (64–100)) than their matched controls (88 (75–96), *p* = 0.03, and 93 (80–100), *p* = 0.04, respectively). In the 4- to 7-year-olds, the median DUX-25 total score was 85 (76–97).

### Behavior

SDQ scores were analyzed in all 31 children. Their total difficulties score (median 10 (IQR 4–14)) did not significantly differ from that of matched controls (6 (3–10), *p* = 0.15; Table 2), and neither did the subscale scores. The total difficulty score was abnormally high in four (13%) children with gastroschisis, compared with seven (11%) matched controls (*p* = 1.00).

### Predictors of cognition and behavior

For cognition, univariable regression analysis revealed that both neonatal intestinal failure and increased parent-perceived child vulnerability were significantly associated with a lower PedsPCF total score (neonatal intestinal failure β − 25.66 (− 49.41 to − 1.91); CVS score β − 2.76 (95% CI − 5.27 to − 0.25); Online Resource 1, Table 1).

For behavior, both older age and SGA were significantly associated with a lower SDS of the SDQ total difficulties score (older age, in years − 0.13 (− 0.24 to − 0.02); SGA − 2.18 (− 3.79 to − 0.57); Online Resource 1, Table 2).

## Discussion

We analyzed parent-reported daily functioning and developmental outcome of children with gastroschisis at school age. Scores on motor function, health status, overall quality of life, and behavior were comparable with those of healthy children. Cognitive problems were reported more frequently in children with gastroschisis, especially in those with neonatal intestinal failure or higher parent-perceived vulnerability.

Previous similar studies have shown contradicting results. Some have reported normal intelligence, motor function, or behavior, whereas others reported intellectual delay, problems regarding motor skills, or behavioral problems (Online Resource 1, Table 3).

The studies that reported normal motor function either included children with omphalocele in their analyses [15] or used a non-standardized questionnaire [16], which complicates comparison of results. A previous study in 16 children with gastroschisis showed normal motor function in only 7 on evaluation with the MABC-2 Test [17]. The difference with our finding of normal scores in 74% may be ascribed to the lower proportion of children born SGA in our study (6% vs. 44%), or to parents overestimating their child’s motor function, or it might imply that the MABC-2 Checklist is less sensitive in diagnosing motor function delay than the MABC-2 Test itself. Our conclusion of normal motor function in children with gastroschisis should, therefore, be regarded with caution.

Children with gastroschisis appeared to be at risk for cognitive problems; PedsPCF scores were lower than those of matched controls, and 16% attended special education, which proportion is higher than in the Dutch reference population (i.e., approximately 5%) [27]. A previous Dutch study in 16 children with gastroschisis found a lower total IQ at school age, and three (19%) attended special education [17]. Two other studies, however, reported normal total IQ in 20 children with gastroschisis at 5 years of age [18] and in 39 children at school age [14]. Remarkably, both studies reported significant problems in working memory [14, 18]. Neonatal critical illness may well have contributed to cognitive problems; exposure to anesthetics, possible hypoxia, inflammation, and stress in early life increase the risk of hippocampal alterations, which may eventually lead to learning problems [11].

Lower PedsPCF scores were associated with increased parent-perceived child vulnerability, which could have several causes. First, parents who perceive their child as highly vulnerable may report more problems, despite normal outcomes at medical evaluation. Early parental counseling and support may positively affect the child’s outcomes as perceived by parents. Second, medical or sociodemographic factors such as intestinal failure or SES could act as confounders, by influencing both child vulnerability and cognitive functioning. Children with intestinal failure scored approximately 26 points (≈ 1 SD) less on the PedsPCF total score than those without intestinal failure. As the prevalence of intestinal failure in the non-responder group was almost twice that in the responder group, we may have underestimated the prevalence of cognitive problems.

In comparison with our study, previous literature showed overall health status in line with normative expectations [5, 28–30]. Our study showed that children with gastroschisis had slightly lower scores on the school functioning subscale of the PedsQL than their matched controls. As median scores differed with only 2 points on a scale of 0–100, we expect this difference not to be clinically relevant.

Although overall quality of life was reported as normal, the DUX-25 subscale scores of physical functioning and home functioning were significantly lower in the gastroschisis group. Negative feelings about physical appearance might be caused by poor physical growth, or by the scar. Home functioning might be impaired by factors associated with the risk of gastroschisis itself, such as teenage pregnancy or maternal mental disorders [31]. However, we acknowledge that these hypotheses are speculative.

Of all children eligible for our study, 18% had been born SGA versus only 6% in the group of parents who returned the questionnaires. Although we should note the very small sample size, being born SGA was significantly associated with behavioral problems. Consequently, the prevalence of behavioral problems in the total gastroschisis population may well be higher. In a previous study including 20 children with gastroschisis, of whom 40% were born SGA, one-third of parents reported behavioral executive problems at 5 years of age [18]. This might still be an underestimation, as we found that older age was significantly associated with behavioral problems, despite the fact that SDS had already been corrected for age.

Strengths of our study include the assessment of outcomes in children beyond the age of 5 years rather than at pre-school age, the comparison of outcomes with those of matched controls, and the availability of neurodevelopmental data at 2 years of age. We used parent-reported outcome measures; since parents are largely responsible for seeking help for their children, we expect our results to be a relevant representation of the need for care in this group. Several limitations need to be addressed. First, while 45% of children with gastroschisis in our cohort were born preterm, we were unable to match controls on GA at birth. A second limitation is the low response rate of 40% and the positive selection bias. Low response rates are a common problem (Online Resource 1, Table 3). As children in the responder group had higher SES, and had experienced less morbidity than non-responders, we may have underestimated the frequency and severity of problems regarding daily functioning. To improve response rates, future studies may limit the number and the length of questionnaires. Based on our outcomes, we would suggest to focus on cognitive functioning and on parent-perceived vulnerability. Additionally, home visits and computerized adaptive testing may help to encourage participation in follow-up studies.

In conclusion, parent-reported outcomes of children with gastroschisis at school age were mainly reassuring. Clinicians and parents should be aware of the higher risk of cognitive problems, especially in those with neonatal intestinal failure or increased parent-perceived vulnerability. We recommend multidisciplinary follow-up at school age of children with neonatal intestinal failure. Early parental counseling and support may positively affect the child’s outcomes as perceived by parents.

## Electronic supplementary material

ESM 1 (PDF 358 kb)

ESM 2 (PDF 283 kb)

ESM 3 (PDF 268 kb)

## Abbreviations

ADHD: Attention deficit hyperactivity disorder
CVS: Child Vulnerability Scale
ISCED: International Standard Classification of Education
LOS: Length of hospital stay
MABC-2: Movement Assessment Battery for Children-Second Edition
MCA: Multiple congenital anomalies
PedsPCF: Pediatric Perceived Cognitive Function
PedsQL: Pediatric Quality of Life Inventory
SDQ: Strengths and Difficulties Questionnaire
SES: Socioeconomic status
SGA: Small for gestational age
TFEF: Time to full enteral feeding
