# Supplementary material for: Gastroschisis at school age: what do parents report?
Source: Eur J Pediatr. 2019 Jul 19;178(9):1405–12. doi: 10.1007/s00431-019-03417-5 (PMC6694033; doi:10.1007/s00431-019-03417-5)
Supplement: Supplementary file 1 — (PDF 358 kb) [file 431_2019_3417_MOESM1_ESM.pdf]

Online Resource 1: tables

Gastroschisis at school age: what do parents report?

European Journal of Pediatrics

Annelieke Hijkoop,[1] André B. Rietman, René M.H. Wijnen, Dick Tibboel, Titia E. Cohen-Overbeek, Joost van Rosmalen, Hanneke IJsselstijn.

[1] Department of Pediatric Surgery and Intensive Care, Erasmus MC – Sophia Children's Hospital, Rotterdam, the Netherlands; a.hijkoop@erasmusmc.nl.

Online Resource 1, Table 1 Univariable linear regression analysis showing possible predictors of the PedsPCF total score (n=23).

	Unstandardized beta	95% confidence interval	p-value
Age at current study (years)	0.19	-3.56 to 3.94	0.92
Child Vulnerability Scale score	-2.76	-5.27 to -0.25	0.03
**Infant clinical data**			
Intoxications during pregnancy			
- Smoking	-17.71	-45.66 to 10.24	0.20
- Recreational drugs	n/a		
Male sex	-9.32	-32.18 to 13.55	0.41
Preterm	-13.82	-36.21 to 8.57	0.21
Small for gestational age	-14.61	-55.29 to 26.08	0.46
Complex gastroschisis	-19.66	-52.97 to 13.65	0.23
Secondary closure	4.55	-21.82 to 30.92	0.72
Multiple congenital anomalies	6.52	-27.84 to 40.88	0.70
Number of procedures under general anesthesia	-5.41	-11.12 to 0.29	0.06
Duration of initial mechanical ventilation (days)	-0.30	-2.10 to 1.51	0.73
Sepsis	-1.63	-26.00 to 22.75	0.89
Length of hospital stay (days)	-0.06	-0.14 to 0.01	0.10
Intestinal failure	-25.66	-49.41 to -1.91	0.04
**Sociodemographic data**			
Maternal age at conception (years)	-0.56	-2.47 to 1.35	0.55
Low status score (<-1)	23.95	-8.78 to 56.68	0.14
Highest parental education level (ISCED level low to high) [$]	-7.89	-22.01 to 6.24	0.26
One caregiver at home	4.86	-20.29 to 30.00	0.69
**Neurodevelopmental data at two years**			
Delayed mental developmental index (<85)	-39.16	-91.36 to 13.04	0.13
Delayed psychomotor developmental index (<85)	20.50	-32.95 to 73.94	0.42

PedsPCF: Pediatric Perceived Cognitive Function; ISCED: International Standard Classification of Education. [$] If paternal education level was unknown, we documented maternal education level as highest parental education level.

Online Resource 1, Table 2 Univariable linear regression analysis showing possible predictors of the SDS of the SDQ total difficulties score (n=31).

	Unstandardized beta	95% confidence interval	p-value
Age at current study (years)	-0.13	-0.24 to -0.02	0.02
Child Vulnerability Scale score	-0.10	-0.21 to 0.00	0.06
**Infant clinical data**			
Intoxications during pregnancy			
- Smoking	-0.46	-1.49 to 0.57	0.37
- Recreational drugs	0.26	-1.32 to 1.85	0.73
Male sex	-0.12	-1.02 to 0.77	0.78
Preterm	0.02	-0.88 to 0.91	0.97
Small for gestational age	-2.18	-3.79 to -0.57	0.01
Complex gastroschisis	0.16	-1.34 to 1.67	0.83
Secondary closure	-0.03	-1.05 to 0.99	0.95
Multiple congenital anomalies	0.03	-1.30 to 1.36	0.96
Number of procedures under general anesthesia	-0.11	-0.38 to 0.15	0.38
Duration of initial mechanical ventilation (days)	-0.04	-0.12 to 0.04	0.29
Sepsis	-0.34	-1.28 to 0.61	0.47
Length of hospital stay (days)	0.00	0.00 to 0.00	0.63
Intestinal failure	-0.25	-1.31 to 0.81	0.63
**Sociodemographic data**			
Maternal age at conception (years)	0.00	-0.07 to 0.08	0.94
Low status score (<-1)	0.44	-0.76 to 1.63	0.46
Highest parental education level (ISCED level low to high) [$]	0.07	-0.57 to 0.71	0.82
One caregiver at home	-0.48	-1.53 to 0.57	0.36
**Neurodevelopmental data at two years**			
Delayed mental developmental index (<85)	-0.01	-1.46 to 1.44	0.99
Delayed psychomotor developmental index (<85)	-0.30	-1.81 to 1.21	0.68

SDS: standard deviation score; SDQ: Strengths and Difficulties Questionnaire; ISCED: International Standard Classification of Education. [$] If paternal education level was unknown, we documented maternal education level as highest parental education level.

Online Resource 1, Table 3 Overview of follow-up studies assessing motor function, cognition, health status, quality of life and/or behavior in children with gastroschisis at school age.

Reference	Outcome	Assessment or questionnaire (self- or parent reported)	Tests	Group	Number of children	Year of birth	Age at follow-up (years)	Normative data	Most important results
Amin et al. 2018 [1]	Health status	Questionnaire (parent)	PedsQL	GS	32	not stated	1-17 (median: 4)	PedsQL parent-report: 88±12	*Health status:* in line with normative expectations (mean 80 (95% CI: 73-86)).
Arnold et al. 2018 [2]	Health status	Questionnaire (parent, telephone)	PedsQL	GS	45/143 (31%)	2009-2012	>3 (median: 5)	PedsQL parent-report: 88±12	*Health status:* in line with normative expectations (median 100 (IQR: 96-100)).
Burnett et al. 2018 [3]	Cognition	Assessment	WPPSI-III	GS	20/35 (57%)	2006-2014	5	TIQ: 100±15	*Cognition*: in line with normative expectations (mean±SD TIQ: 100±10).
	Behavior	Questionnaires (parent)	BRIEF-P and BASC-2					Elevated scores: BRIEF-P: 7%, BASC-2: 16%	*Behavior*: a substantial number of children fell in the elevated range across BRIEF-P scales (e.g. over 40% scored abnormal for the working memory scale).
Lap et al. 2017 [4]	Motor function	Assessment	M-ABC	GS	16	1999-2006	5-13 (median: 9)	Normal: 85%; at risk: 10%; problem: 5%	*Motor function*: significantly poorer than matched controls on all M-ABC scores, especially on fine motor skills.
	Cognition	Assessment	WISC-III					TIQ: 100±15	*Cognition:* significantly lower TIQ and verbal IQ than matched controls (mean±SD TIQ: 92±13; median verbal IQ: 95 (IQR: 88-100)). 19% received special education, 58% had repeated a grade.
	Behavior	Questionnaires (parent)	CBCL<br>BRIEF					Normal: 85%; at risk: 10%; problem: 5%	*Behavior*: not significantly different from matched controls (normal: 75%, borderline: 6%, abnormal: 19%).
Rankin et al. 2016 [5]	Health status	Questionnaire (both)	KIDSCREEN	GS	10/53 (19%)	not stated	8-11 (median 10)	KIDSCREEN self-report: 10 domains	*Health status*: all domains in line with normative expectations (e.g. mean±SD physical well-being:

								(between 45±12 and 53±10)	54±12). All children described health as good/very good or excellent.
Carpenter et al. 2016 [6]	Health status	Questionnaire (parent)	PedsQL	GS	28/119 (24%)	2005-2011	>2 (mean: 6)	PedsQL parent-report: 88±12	*Health status:* in line with normative data (mean±SD simple GS: 82±20, complex GS: 79±20)
Harris et al. 2016 [7]	Cognition	Assessment	WPPSI-III (if <6 years old) or WISC-IV	GS	39/99 (39%)		5-17 (median: 10)	TIQ: 100±15	*Cognition:* in line with normative data (mean±SD: 98±11)
	Behavior	Questionnaire (parent)	SDQ					SDQ total difficulties score: 7±6	*Behavior*: significantly more problems than healthy children (mean±SD: 11±7).
Giúdici et al. 2016 [8]	Motor function / cognition	Assessment	PRUNAPE	GS	17/62 (27%)	2002-2013	6	n/a	*Motor function / cognition:* normal in 35%. 35% attended a special school.
Hamrick et al. 2010 [9]	Cognition	Linkage with education files public schools	n/a	GS	134 (128 isolated, 6 non-isolated)	1982-2001	Ever received special education between 3-10	Use of special education services: 8%	*Cognition*: use of special education services in line with normative data for isolated gastroschisis (6%), and higher for non-isolated gastroschisis (50%).
Van der Cammen-van Zijp et al. 2010 [10]	Motor function	Assessment	M-ABC	GS+OC	24/33 (73%)	1999-2003	5	Normal: 85%; at risk: 10%; problem: 5%	*Motor function*: in line with normative data (normal: 79%, at risk: 13%, problem: 8%).
Henrich et al. 2008 [11]	Motor function	Questionnaire (not stated)	not standardized	GS	22/40 (55%)	1994-2004	1-10 (median 6)	n/a	*Motor function*: 9% problems with physical exercise, one child felt restricted in sporting activities.
	Cognition	Questionnaire (not stated)	not standardized					n/a	*Cognition*: 77% had attended kindergarten or school at usual age.
Ginn-Pease et al. 1991 [12]	Cognition	Assessments	WISC-R<br>WJ-R	GS+OC	22/93 (24%)	1972-1981	6-16 (mean: 11)	TIQ: 100±15	*Cognition*: in line with normative data (mean±SD: 100±16).
	Behavior	Questionnaires (parent)	VABS<br>CBCL					VABS ≥ 85.<br>ACBC < 63	*Behavior*: mean scores within normal range (VABS: 95±16; CBCL: 57±10); however, 18%

	exceeded the 90th percentile on the CBCL.

GS: gastroschisis; OC: omphalocele; CI: confidence interval; IQR: interquartile range; TIQ: total intelligence quotient; SD: standard deviation.

PedsQL: Pediatric Quality of Life Inventory;
WPPSI-III: Wechsler Preschool and Primary Scale of Intelligence-3rd edition;
BRIEF: Behavior Rating Inventory of Executive Functioning (-P: preschool version);
BASC-2: Behavior Assessment System for Children – 2nd edition (preschool);
M-ABC: Movement Assessment Battery for Children;
WISC: Wechsler Intelligence Scale for Children (-R: revised; -III: 3rd edition; IV: 4th edition);
CBCL: Child Behavior Checklist;
SDQ: Strengths and Difficulties Questionnaire;
PRUNAPE: Prueba Nacional de Pesquisa [Argentine screening instrument];
WJ-R: Woodcock-Johnson Psycho-Educational Battery-revised;
VABS: Vineland Adaptive Behavior Scale.

References

1. Amin R, Knezevich M, Lingongo M, Szabo A, Yin Z, Oldham KT, Calkins CM, Sato TT, Arca MJ (2018) Long-term Quality of Life in Neonatal Surgical Disease. Ann Surg 268:497-505
2. Arnold HE, Baxter KJ, Short HL, Travers C, Bhatia A, Durham MM, Raval MV (2018) Short-term and family-reported long-term outcomes of simple versus complicated gastroschisis. J Surg Res 224:79-88
3. Burnett AC, Gunn JK, Hutchinson EA, Moran MM, Kelly LM, Sevil UC, Anderson PJ, Hunt RW (2018) Cognition and behaviour in children with congenital abdominal wall defects. Early Hum Dev 116:47-52
4. Lap CC, Bolhuis SW, Van Braeckel KN, Reijneveld SA, Manten GT, Bos AF, Hulscher JB (2017) Functional outcome at school age of children born with gastroschisis. Early Hum Dev 106-107:47-52
5. Rankin J, Glinianaia SV, Jardine J, McConachie H, Borrill H, Embleton ND (2016) Measuring self-reported quality of life in 8- to 11-year-old children born with gastroschisis: Is the KIDSCREEN questionnaire acceptable? Birth Defects Res A Clin Mol Teratol 106:250-256
6. Carpenter JL, Wiebe TL, Cass DL, Olutoye OO, Lee TC (2016) Assessing quality of life in pediatric gastroschisis patients using the Pediatric Quality of Life Inventory survey: An institutional study. J Pediatr Surg 51:726-729
7. Harris EL, Hart SJ, Minutillo C, Ravikumara M, Warner TM, Williams Y, Nathan EA, Dickinson JE (2016) The long-term neurodevelopmental and psychological outcomes of gastroschisis: A cohort study. J Pediatr Surg 51:549-553
8. Giudici L, Bokser VS, Maricic MA, Golombek SG, Ferrario CC (2016) Babies born with gastroschisis and followed up to the age of six years faced long-term morbidity and impairments. Acta Paediatr 105:e275-280
9. Hamrick SE, Strickland MJ, Shapira SK, Autry A, Schendel D (2010) Use of special education services among children with and without congenital gastrointestinal anomalies. Am J Intellect Dev Disabil 115:421-432
10. van der Cammen-van Zijp MH, Gischler SJ, Mazer P, van Dijk M, Tibboel D, Ijsselstijn H (2010) Motor-function and exercise capacity in children with major anatomical congenital anomalies: an evaluation at 5 years of age. Early Hum Dev 86:523-528
11. Henrich K, Huemmer HP, Reingruber B, Weber PG (2008) Gastroschisis and omphalocele: treatments and long-term outcomes. Pediatr Surg Int 24:167-173
12. Ginn-Pease ME, King DR, Tarnowski KJ, Green L, Young G, Linscheid TR (1991) Psychosocial adjustment and physical growth in children with imperforate anus or abdominal wall defects. J Pediatr Surg 26:1129-1135
