# Supplementary material for: Gastroschisis at school age: what do parents report?
Source: Eur J Pediatr. 2019 Jul 19;178(9):1405–12. doi: 10.1007/s00431-019-03417-5 (PMC6694033; doi:10.1007/s00431-019-03417-5)
Supplement: Supplementary file 2 — (PDF 283 kb) [file 431_2019_3417_MOESM2_ESM.pdf]

Online Resource 2: description of questionnaires

Gastroschisis at school age: what do parents report?

European Journal of Pediatrics

Annelieke Hijkoop,[1] André B. Rietman, René M.H. Wijnen, Dick Tibboel, Titia E. Cohen-Overbeek, Joost van Rosmalen, Hanneke IJsselstijn.

[1] Department of Pediatric Surgery and Intensive Care, Erasmus MC – Sophia Children's Hospital, Rotterdam, the Netherlands; a.hijkoop@erasmusmc.nl.

Description of questionnaires

We used Dutch versions of all questionnaires. All questionnaires were parent-reported. If two answers were selected for one question, we documented the most unfavorable score.

Background

Description: We asked caregivers to report the following background information: child's living situation (e.g. with biological parents or in a foster family, presence of two caregivers, number of children), maternal and paternal education levels (based on the International Standard Classification of Education 2011) [1], medical (use of medication, hospital admissions, use of medical aids such as a wheelchair or parenteral nutrition), educational (e.g. regular or special, grade repetition, learning difficulties, need of extra help at school), social-emotional functioning (presence of behavioral or emotional problems), and main language spoken at home.

Child vulnerability

<u>Child Vulnerability Scale (CVS) [2,3]</u>

Description: The CVS is an 8-item questionnaire on parental perceptions of their child's vulnerability. Each item states a problem, for example 'my child gets more colds than other children I know'. Answers vary from strongly disagree (=0) to strongly agree (=3) on a 4-point Likert scale. Total scores range from 0-24; higher scores reflect higher perceived vulnerability. We used a cut-off of ≥10 for high perception of vulnerability.

Validated: This questionnaire has been validated for Dutch children aged 5-18 years [2].

Motor function

<u>Movement Assessment Battery for Children- Second Edition (MABC-2) Checklist [4-6]</u>

Description: The M-ABC 2 Checklist is aimed at evaluating motor problems in daily life. Section A measures movement in a static (or predictable) environment; section B measures movement in a dynamic (or unpredictable) environment. Both sections consist of 15 items. Each of the 30 items states a skill, for

example 'rides a bicycle without stabilizers'. The parent indicates to what extent the child is able to do this, varying from very well (=0) to not close (=3). Scores are reported using a Traffic Light color system, corrected for age, with high scores representing poor performance. 'Green zone' indicates a score within the normal range (< 85th centile); 'amber zone' means that the child is at risk for motor problems (85th-94th centile), and a score in the 'red zone' indicates a high possibility of serious motor problems (≥ 95th centile).

Validated: This questionnaire has been validated for Dutch children aged 3-16 years [6]. As no Dutch reference norms exist for 17-year old children, these children were scored according to reference norms for 16-year olds.

Cognition

Pediatric Perceived Cognitive Function (PedsPCF) questionnaire [7]

Description: The PedsPCF assesses the child's cognitive functioning as perceived by the parent, referring to the past four weeks. Each item reflects a problem, for example 'forgets things easily'. Answers vary from very much/all of the time (=1) to not at all/none of the time (=5) on a 5-point Likert scale. Based on preliminary results of the collection of Dutch reference data, we used only the first 30 items of the PedsPCF rather than the full-length PedsPCF (which counts 43 items), and we used the following cut-offs of ≤-1 standard deviation (SD): 102 (7-12 years), 104 (13-18 years). Total scores range from 30-150; higher scores reflect better cognitive functioning.

Validated: This questionnaire has been validated for Dutch children aged 7-18 years [8].

Health status

Pediatric Quality of Life Inventory (PedsQL) [9]

Description: The PedsQL is an instrument for measuring health status in children and adolescents. It consists of four subscales: physical (8 items), emotional (5 items), social (5 items) and school functioning (5 items). Each item reflects a problem, for example 'problems with running'. Answers vary from never (=0) to almost always (=4) on a 5-point Likert scale. Each answer is reversed scored and rescaled to a 0-100 scale

(0=100, 4=0). Total scores range from 0-100; higher scores reflect better quality of life. We used the version that referred to the past month.

Validated: This questionnaire has been validated for Dutch children aged 5-18 years [10].

Quality of life

DUX-25

Description: The DUX-25 is a visual health-related quality of life questionnaire. Each question evaluates the child's feelings in daily life, for example 'your child often feels …'. It consists of four subscales: physical (6 items), emotional (7 items), social (7 items) and home functioning (5 items). Answers are scored on a happy-to-sad faces scale by use of smileys. These smileys visualize a 5-point Likert scale, ranging from sad (=0) to happy (=100). Total scores range from 0-100; higher scores reflect better quality of life.

Validated: Dutch reference data are currently being analysed (age 8-17 years).

Behavior

Strengths and Difficulties Questionnaire (SDQ) [11]

Description: The SDQ covers the most important domains of child psychopathology and personal strengths. It consists of five subscales: emotional symptoms, conduct problems, hyperactivity-inattention, peer problems, and prosocial behavior. Each item is scored on a 3-point Likert scale; answer vary from not true (=0) to certainly true (=2). Higher scores reflect more difficulties, except for the prosocial scale where higher scores reflect strengths. All but the prosocial behavior subscale scores are summed to generate a total difficulties score. Total scores range from 0-40. The total difficulties score was categorized into 'normal' or 'abnormal' using age-dependent cut-off values [11].

Validated: This questionnaire has been validated for Dutch children aged 2-18 years [11]. In children aged <6 years, no SD scores or cut-off values were available for 'conduct problems' and 'peer problems' due to insufficient internal consistency of these subscales in this age group.

References

1. (2012) International Standard Classification of Education ISCED 2011. UNESCO Institute for Statistics, Montreal, Canada
2. Houtzager BA, Moller EL, Maurice-Stam H, Last BF, Grootenhuis MA (2015) Parental perceptions of child vulnerability in a community-based sample: Association with chronic illness and health-related quality of life. J Child Health Care 19:454-465
3. Forsyth BW, Horwitz SM, Leventhal JM, Burger J, Leaf PJ (1996) The child vulnerability scale: an instrument to measure parental perceptions of child vulnerability. J Pediatr Psychol 21:89-101
4. Brown T, Lalor A (2009) The Movement Assessment Battery for Children--Second Edition (MABC-2): a review and critique. Phys Occup Ther Pediatr 29:86-103
5. Henderson SE, Sugden DA, Barnett AL (2007) Movement assessment battery for children-second edition [Movement ABC-2]. Pearson, London, United Kingdom
6. Smits-Engelsman B (2010) Dutch Manual Movement Assessment Battery for Children-2. Pearson, Amsterdam, The Netherlands
7. Lai JS, Butt Z, Zelko F, Cella D, Krull KR, Kieran MW, Goldman S (2011) Development of a parent-report cognitive function item bank using item response theory and exploration of its clinical utility in computerized adaptive testing. J Pediatr Psychol 36:766-779
8. Marchal JP, de Vries M, Conijn J, Rietman AB, H IJ, Tibboel D, Haverman L, Maurice-Stam H, Oostrom KJ, Grootenhuis MA (2019) Pediatric Perceived Cognitive Functioning: Psychometric Properties and Normative Data of the Dutch Item Bank and Short Form. J Int Neuropsychol Soc:1-12
9. Varni JW, Seid M, Kurtin PS (2001) PedsQL 4.0: reliability and validity of the Pediatric Quality of Life Inventory version 4.0 generic core scales in healthy and patient populations. Med Care 39:800-812
10. Engelen V, Haentjens MM, Detmar SB, Koopman HM, Grootenhuis MA (2009) Health related quality of life of Dutch children: psychometric properties of the PedsQL in the Netherlands. BMC Pediatr 9:68
11. Maurice-Stam H, Haverman L, Splinter A, van Oers HA, Schepers SA, Grootenhuis MA (2018) Dutch norms for the Strengths and Difficulties Questionnaire (SDQ) - parent form for children aged 2-18 years. Health Qual Life Outcomes 16:123
