# Supplementary material for: Gastroschisis at school age: what do parents report?
Source: Eur J Pediatr. 2019 Jul 19;178(9):1405–12. doi: 10.1007/s00431-019-03417-5 (PMC6694033; doi:10.1007/s00431-019-03417-5)
Supplement: Supplementary file 3 — (PDF 268 kb) [file 431_2019_3417_MOESM3_ESM.pdf]

Online Resource 3: description of matched controls

Gastroschisis at school age: what do parents report?

European Journal of Pediatrics

Annelieke Hijkoop,[1] André B. Rietman, René M.H. Wijnen, Dick Tibboel, Titia E. Cohen-Overbeek, Joost van Rosmalen, Hanneke IJsselstijn.

[1] Department of Pediatric Surgery and Intensive Care, Erasmus MC – Sophia Children's Hospital, Rotterdam, the Netherlands; a.hijkoop@erasmusmc.nl.

Description of matched controls

We obtained matched controls from three different datasets, as described below. Controls were matched for age (maximum difference of one year), gender, and maternal education level (low, middle, or high; based on the International Standard Classification of Education 2011 [1]). Controls were selected randomly using an online randomizer. If the maternal education level of a case was unknown, this case was matched with two controls with middle maternal education level.

Cognition

<u>Pediatric Perceived Cognitive Function (PedsPCF) questionnaire</u>

Matched controls were obtained from a study that collected Dutch normative data for the PedsPCF [2]. A general population sample of parents and their children had been approached through research agency Kantar TNS in January 2016. This study included children with a chronic health condition, such as asthma or diabetes mellitus. This study used online questionnaires. We used the parent-reported data.

Health status and quality of life

<u>Pediatric Quality of Life Inventory (PedsQL) and DUX-25</u>

Matched controls were obtained from a study that collected Dutch normative data for the PedsQL (4-17 years) and the DUX-25 (8-17 years) (publications in preparation). Children with a chronic health condition had been excluded from this study; those with attention deficit hyperactivity disorder had been included. Online questionnaires had been sent to caregivers and their children, who were recruited via primary and secondary schools in the Netherlands from April 2015 till March 2016. We used the parent-reported data.

Behavior

Strengths and Difficulties Questionnaire (SDQ)

Matched controls were obtained from the database of Maurice-Stam and coworkers [3]. A general population sample of parents had been approached through research agency Kantar TNS in November and December 2014.

References

1. (2012) International Standard Classification of Education ISCED 2011. UNESCO Institute for Statistics, Montreal, Canada
2. Marchal JP, de Vries M, Conijn J, Rietman AB, H IJ, Tibboel D, Haverman L, Maurice-Stam H, Oostrom KJ, Grootenhuis MA (2019) Pediatric Perceived Cognitive Functioning: Psychometric Properties and Normative Data of the Dutch Item Bank and Short Form. J Int Neuropsychol Soc:1-12
3. Maurice-Stam H, Haverman L, Splinter A, van Oers HA, Schepers SA, Grootenhuis MA (2018) Dutch norms for the Strengths and Difficulties Questionnaire (SDQ) - parent form for children aged 2-18 years. Health Qual Life Outcomes 16:123
